# Linking Phospho-Gonadotropin Regulated Testicular RNA Helicase (GRTH/DDX25) to Histone Ubiquitination and Acetylation Essential for Spermatid Development During Spermiogenesis

**DOI:** 10.3389/fcell.2020.00310

**Published:** 2020-05-15

**Authors:** Raghuveer Kavarthapu, Rajakumar Anbazhagan, Ashish K. Sharma, Joseph Shiloach, Maria L. Dufau

**Affiliations:** ^1^Section on Molecular Endocrinology, Division of Developmental Biology, Eunice Kennedy Shriver National Institute of Child Health and Human Development, National Institutes of Health, Bethesda, MD, United States; ^2^Biotechnology Core Laboratory, National Institute of Diabetes and Digestive and Kidney Diseases, National Institutes of Health, Bethesda, MD, United States

**Keywords:** phospho-GRTH/DDX25, spermatogenesis, round spermatids, histones, ubiquitination, acetylation

## Abstract

GRTH/DDX25 is a testicular RNA helicase expressed in germ cells that plays a crucial role in completion of spermatogenesis. Previously, we demonstrated a missense mutation (R^242^H) of GRTH gene in Japanese infertile patients (5.8%) with non-obstructive azoospermia. This mutation upon expression in COS-1 cells revealed absence of the 61 kDa phosphorylated GRTH in cytoplasm and the presence of the 56 kDa non-phosphorylated GRTH in the nucleus. GRTH knock-in (KI) mice carrying the human GRTH (R^242^H) mutation, lack phosphorylated GRTH, and sperm due to failure of round spermatid elongation during spermiogenesis. To determine the impact of phosphorylated GRTH on molecular events/pathways participating in spermatid development during spermiogenesis, we analyzed transcriptome profiles obtained from RNA-Seq of germ cells from KI and WT mice. RNA-Seq analysis of 2624 differentially expressed genes revealed 1404 down-regulated and 1220 up-regulated genes in KI mice. Genes relevant to spermatogenesis, spermatid development and spermatid differentiation were significantly down-regulated. KEGG enrichment analysis showed genes related to ubiquitin-mediated proteolysis and protein processing in endoplasmic reticulum pathway genes were significantly down-regulated while the up-regulated genes were found to be involved in Focal adhesion and ECM-receptor interaction pathways. Real-Time PCR analysis confirmed considerable reduction in transcripts of ubiquitination related genes *Ube2j1*, *Ube2k*, *Ube2w*, *Rnf8*, *Rnf133*, *Rnf138, Cul3* and increased expression of *Ccnd2*, *Col1a*, *Lamb1*, *Cav1*, *Igf1*, *Itga9* mRNA’s in KI mice compared to WT. Also, marked reduction in protein expression of UBE2J1, RNF8, RNF138 (ubiquitination network), MOF (histone acetyltransferase), their modified Histone substrates (H2AUb, H2BUb) and H4Ac, H4K16Ac were observed in KI mice. GRTH-IP mRNA binding studies revealed that *Rnf8* and *Ube2J1* mRNAs from WT mice associated with GRTH protein and the binding is greatly impaired in the KI mice. Immunohistochemistry analysis showed significantly reduced expression of RNF8, MOF, H4Ac and H4K16Ac in round spermatids of KI mice. Absence of phosphorylated GRTH impairs UBE2J1, RNF8 and MOF-dependent histone ubiquitination and acetylation essential for histone replacement, chromatin condensation and spermatid elongation during spermiogenesis.

## Introduction

Gonadotropin-regulated testicular RNA helicase (GRTH/DDX25) belongs to DEAD-box protein family of RNA helicases explicitly found in the testis and is transcriptionally regulated by gonadotropins via paracrine androgen action (Tang et al., 1999; Sheng et al., 2003; Dufau and Tsai-Morris, 2007; Dufau and Kavarthapu, 2019). GRTH/DDX25 is expressed in Leydig cells, meiotic spermatocytes and haploid spermatids (Sheng et al., 2003; Dufau and Tsai-Morris, 2007; Dufau and Kavarthapu, 2019). It is involved in multiple functions as a post-transcriptional controller of specific genes in germ cells during spermatogenesis. In germ cells GRTH is an integral constituent of messenger ribonuclear protein complex, that transports specific mRNAs from the nucleus to cytoplasmic sites including chromatoid bodies for storage prior to their translation during spermatogenesis. Chromatoid bodies (CB) are non-membranous organelles residing in the cytoplasm of round spermatids (RS) where specific mRNAs are stored and later processed during spermiogenesis (Tsai-Morris et al., 2004b; Dufau and Tsai-Morris, 2007; Dufau and Kavarthapu, 2019). We have shown that GRTH binds to actively translating polyribosomes, where may play an important role in translation of specific genes in germ cells like transition proteins (TP1/TP2), protamines (PRM1/PRM2), phosphoglycerate kinase and testicular angiotensin converting enzyme (Sheng et al., 2006; Sato et al., 2010; Tsai-Morris et al., 2012). The GRTH gene with TATA-less promotor has many transcriptional start sites and it is transcriptionally regulated by Sp1/Sp3 (Tsai-Morris et al., 2004a). We have previously reported two forms of GRTH protein in germ cells, a 61 kDa phosphorylated species exclusively found in the cytoplasm and a 56 kDa non-phosphorylated species in the nucleus. The 61 kDa phosphorylated GRTH (p-GRTH) protein in the cytoplasm participates in shuttling of specific mRNAs in and out of the CB and also associates with polyribosomes for translation. The 56 kDa non-p-GRTH protein is involved in the export of mRNAs from nucleus to cytoplasm (Sheng et al., 2006; Dufau and Tsai-Morris, 2007) and has also being proposed to participate in transcriptional events of Drosha-DGCR8 complex (Dai et al., 2011).

GRTH knock-out mice are infertile with complete loss of elongating spermatids (ES) and sperm due to spermatogenic arrest at step 8. In the RS of these null mice, the size of the CB was markedly reduced indicating either decrease or loss of mRNAs essential for progression of spermiogenesis (Tsai-Morris et al., 2004b; Sheng et al., 2006). GRTH is the only member of the DEAD-box helicase family known to be transcriptionally up-regulated by gonadotropins (luteinizing hormone/human chorionic gonadotropin). In Leydig cells, luteinizing hormone through stimulation of androgen production induces GRTH transcription in an autocrine manner through an androgen responsive element present in the proximal region of the GRTH gene (Tsai-Morris et al., 2010; Villar et al., 2012). In germ cells, where GRTH expression is both cell- and stage-specific, paracrine actions of androgen via its receptors in Sertoli cells increase transcription of GRTH in germ cells (Kavarthapu et al., 2013).

Our previous studies identified a missense heterozygous mutation of Arg to His at position 242 (R^242^H) in GRTH gene in 5.8% of Japanese men with non-obstructive azoospermia (Tsai-Morris et al., 2007). *In vitro* experiments performed by overexpressing the human mutant GRTH construct in COS-1 cells revealed the loss of the cytoplasmic 61 kDa p-GRTH species, while maintaining the expression of 56 kDa non-phospho form (Tsai-Morris et al., 2007). Also, we established that GRTH was phosphorylated by Protein Kinase A (Sheng et al., 2006). Subsequently, we created transgenic GRTH Knock-In (KI) mice bearing the hGRTH gene with the R^242^H mutation which lack the 61 kDa cytoplasmic p-GRTH form (Kavarthapu et al., 2019). Homozygous GRTH-KI mice are infertile with absence of mature sperm due to failure of RS to elongate while exhibited normal mating behavior. In these KI mice loss of p-GRTH has significant effects on the levels of mRNA and protein of TP2, PRM2 and TSSK6 (Kavarthapu et al., 2019). To understand mechanistically the impact of p-GRTH on the round spermatids elongation process we investigated differential expression of genes and compared transcriptome profiles obtained from germ cells of KI and WT using Illumina RNA-Seq. This study indicates the essential role of p-GRTH/DDX25 in UBE2J1 and RNF8 dependent histone modification during spermiogenesis.

## Materials and Methods

### Animals and Preparation of Germ Cells

The generation of GRTH-KI mice carrying human GRTH gene with R^242^H mutation were described previously (Kavarthapu et al., 2019). Homozygous KI mice were obtained by crossing heterozygous KI male mice either with heterozygous or homozygous KI female mice. KI mice were genotyped using two primers sets, KI-F1/KI-R1 and KI-F2/KI-R2 (Supplementary Table S1) to detect targeted and mice GRTH alleles, respectively. Transgenic animals were maintained at 22°C in a pathogen free, light controlled environment with an alternating light–dark cycle. All animal studies were performed as per the guidelines of National Institute of Child Health and Human Development Animal Care and Use Committee. Germ cells were prepared individually from five mice (45 days old) each for WT and KI by collagenase-trypsin dispersion. Testes were decapsulated and the seminiferous tubules were treated with collagenase in M199 medium containing 0.1% bovine serum albumin (BSA) for 15 min. The collagenase treated tubules were minced and incubated in M199 with 0.1% BSA and 0.1% trypsin for 15 min at 35°C in rotation at 100 rpm to obtain dispersed cell suspension. After trypsin treatment 0.02% of trypsin inhibitor (Sigma) was added to the sample and filtered through 300 μm mesh strainer and glass wool and then passed through 100 and 40 μm cell strainer (Millipore) to obtain germ cell fraction (spermatogonia, spermatocytes and spermatids) which was pelleted and washed with cold PBS for total RNA isolation.

### Total RNA Preparation and Library Construction for RNA-Seq

Total RNA was prepared from germ cells isolated from WT and KI testis using Qiagen RNeasy mini kit (Qiagen, CA). RNA concentration and quality were evaluated using the Agilent 2100 Bioanalyzer system (Agilent Technologies, CA, United States). mRNA was then extracted from total RNA using oligo (dT) method and RNA-Seq library was constructed according to a standard protocol provided by Illumina. Sequencing was performed for a paired-end 150 bp on the Illumina HiSeq 2500 by Novogene Bioinformatics Institute (Beijing, China). The raw RNA-seq reads have been deposited to the NCBI^1^ GEO database with GEO accession number GSE145047.

### Transcriptome Analysis and Identification of Differential Gene Expression

RNA-seq reads were first trimmed for adapters and then aligned to mouse mm10 reference genome sequences using STAR in Patek Flow online software^2^ for next-generation sequencing analysis. The aligned reads were quantitated to Partek E/M (mm10 RefSeq_v91) annotation model to obtain gene counts. Principal component analysis (PCA) was done on gene counts to determine the variability in the data set. Differentially expressed genes (DEGs) between WT and KI conditions were generated with DESeq2 (fold change ≤−1.5 or ≥1.5; *P* < 0.05, FDR < 0.05) in Partek Flow.

### Protein-Protein Interaction (PPI) Network Analysis

STRING^3^ is the online database used for known and predicted protein-protein interactions to analyze the interaction relationships between proteins in our study. To visualize the interactions among the down-regulated DEGs, STRING tool was used to construct a PPI network with a interacting confidence scores of >0.4 designated as the cutoff. Cytoscape 3.7.2 software was employed to analyze the PPI network by Network Analyzer plugin. cytoHubba plugin in cytoscape was used to extract the top 20 hub genes from the PPI network based on maximal clique centrality (MCC) algorithm.

### Enrichment Analysis of DEGs

To identify biological functional of DEGs, Gene Ontology (GO) enrichment analysis was done, and the data was visualized using DAVID^4^ online tool. GO functional analysis of DEGs were sub-divided into three groups: biological process (BP), molecular function (MF) and cellular component (CC). A *P*-value of less than 0.05 and gene count >5 was set as the cutoff. KEGG pathway analysis was carried out on up-regulated and down-regulated DEGs separately using clusterProfiler package in R-Studio 3.6.1 to identify the crucial pathways with *P*-value < 0.05.

### Real-Time PCR Analysis for the Validation of RNA-Seq Results

Real-time quantitative reverse transcription PCR (qRT-PCR) was used to validate the DEGs obtained from RNA-Seq analysis. Total RNA (1 μg) prepared for RNA-Seq library was Reverse transcribed using the SuperScript III first-strand synthesis SuperMix (ThermoFisher Scientific, MA). qRT-PCR was then performed with Fast SYBR green master mix in a final volume of 20 μL. All PCR reactions were done in triplicates in a 7500 Fast Real-Time PCR machine (Applied Biosystems, CA). Cycle threshold (Ct) values were normalized to β-actin as reference gene, and Relative quantification of transcripts was performed using the comparative 2^–ΔΔ*Ct*^ method. The primer sets used in this analysis are available in Supplementary Table S1.

### Western Blot Analysis

Total protein lysates from testis of WT and KI mice were extracted using RIPA lysis buffer (Upstate, Temecula, CA, United States) containing Halt protease and phosphatase inhibitor cocktail (ThermoFisher Scientific, MA, United States). The supernatants of lysates centrifuged at 2,500 *g* for 5 min were used to estimate the protein concentration by the Bradford assay (Bio-Rad Laboratories, CA, United States). These lysates (100 μg) were resolved in 4–12% Bis-Tris gel (Invitrogen) and transferred onto nitrocellulose membrane using Iblot 2 (ThermoFisher Scientific, MA, United States). Membranes blocked with 5% skimmed milk powder in PBS were incubated with the corresponding primary antibodies (Supplementary Table S2) at 1:1000 dilution. After the incubation of primary antibodies and washing steps, the membranes were incubated with the respective secondary antibodies conjugated with poly-HRP at 1:2000 dilution. Immunosignals were detected by a Tanon High-sig ECL Western Blotting Substrate system (ABclonal, MA, United States).

### Immunohistochemistry

Paraformaldehyde fixed cross-sections were deparaffinized and rehydrated in a graded series of ethyl alcohol and distilled water. Antigen retrieval for 10 min was performed using Tris-EDTA buffer pH 9.0 before blocking with horse serum. The slides were incubated overnight at 4°C with 1:250 dilution of primary antibodies of RNF8, MYST/MOF, H4-Ac, H4K16-Ac, and Ube2J1 or rabbit IgG isotype control. Subsequently, sections were incubated secondary antibody conjugated with horseradish peroxidase polymer for 1 h. (Invitrogen, Frederick, MD, United States). After washing the sections with PBS, diaminobenzidine as chromagen and hydrogen peroxide as substrate were added to develop the brown color for visualization. Then the sections were counterstained with hematoxylin.

### Immunoprecipitation (IP) of GRTH-RNA Complex From Testis

Total protein extracts (0.5 mg) isolated from germ cells of WT and GRTH-KI mice using RIPA lysis buffer (Upstate, Temecula, CA, United States) were initially incubated with 50 μl of protein A/G Plus-agarose beads and 1 μg of rabbit IgG in IP binding buffer (ThermoFisher Scientific, MA) with gentle agitation for 30 min at 4°C. Supernatants were incubated with 4 μg affinity-purified anti-GRTH rabbit polyclonal antibody for overnight at 4°C to co-immunoprecipitate the GRTH-ribonucleoprotein complex. 50 μl of protein A-agarose beads was added and incubated for 2 h at 4°C. The GRTH-ribonucleoprotein complex bound to protein A-agarose was washed three times with IP binding buffer. The RNA from this GRTH-ribonucleoprotein complex was isolated by phenol/chloroform/isoamyl alcohol (25:24:1, v/v; Invitrogen). First-strand cDNA was prepared using SuperScript III first-strand synthesis kit (ThermoFisher Scientific, MA) and Real-Time PCR was performed with Fast SYBR green using specific set of primers for RNF8 and UBE2J1 (Supplementary Table S1) in a 7500 Fast Real-Time PCR machine (Applied Biosystems, CA, United States).

### Statistical Analysis

Data was analyzed using Prism 8.0 statistical software (GraphPad Software Inc, San Diego, CA, United States). Differences between KI and WT groups were determined by two-tailed Students *t*-test. The error bars represent the standard error mean (SEM) with *P* < 0.05 was considered statistically significant.

## Results

### Summary of RNA-Seq Data

**FIGURE 1:**
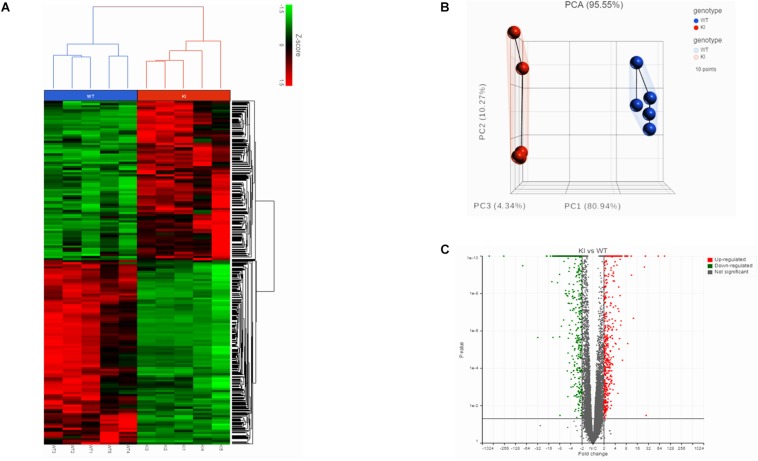
Transcriptome analysis of germ cells from KI and WT mice. **(A)** Hierarchical clustered heatmap showing the expression patterns of significantly (fold change ≤–1.5 or ≥1.5; *p* < 0.05) DEGs. The red boxes indicate the up-regulated genes, and the green boxes indicates down-regulated genes. Colored bar represents the expression levels. **(B)** PCA plot showing clustered samples based on the KI and WT genotype. Each point represents replicate sample for each genotype. **(C)** Volcano plot (fold change vs *P*-value) showing 2624 DEGs from KI and WT group (*n* = 5 per group). Red dots represent up-regulated genes and green dots down-regulated genes in KI vs WT.

We initially performed RNA-Seq to analyze the transcriptomes of mouse germ cells from KI and WT groups. In total, we obtained 93 and 111.2 million reads from RNA-Seq libraries made from the KI and WT mice germ cells, respectively. The average read quality (Phred33 score) was 35.8 and more than 95% of reads could be mapped to the mice reference genome (Supplementary Table S3). Separation of the KI and WT genotypes was evident in the Hierarchically clustered heatmap shown in the expression profiles of DEGs (Figure 1A). PCA analysis revealed clustering of five biological replicates in each group clearly demonstrated the differences between KI and WT (Figure 1B).

### Identification of Differentially Expressed Genes (DEGs) and Functional Variation

**FIGURE 2:**
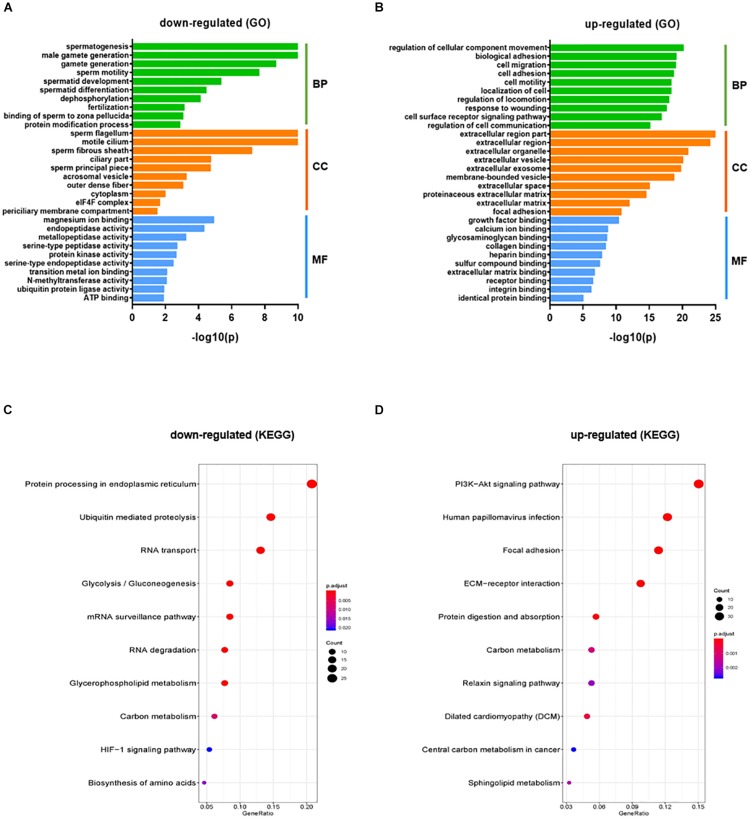
GO and KEGG pathway enrichment analysis of DEGs. GO functional analysis (Top 10 GO terms) for down-regulated DEGs **(A)** and up-regulated DEGs **(B)** in KI vs WT mice were grouped into different functional categories: biological process (BF), cellular component (CC) and molecular function (MF). **(B)** Representative dot plot of top 10 significantly (*P* < 0.05) enriched KEGG pathways for down-regulated **(C)** and up-regulated genes **(D)**. The *x*-axis represents gene ratio = count/set size. Dot size represents the number of genes and the color bar represents the padj-value.

The gene count data was used to analyze differences in gene expression by DESeq2. A total of 2624 DEGs were identified, including 1220 significantly up-regulated and 1404 significantly down-regulated genes between KI and WT groups (Supplementary Table S4). Volcano plot for DEGs are shown in Figure 1C. To further analyze and classify the biological function of DEGs, we performed GO functional analysis using DAVID software and KEGG pathway enrichment analysis was done using R-studio. GO analysis of DEGs were sub-divided into three categories: biological process (BP), cellular component (CC) and molecular function (MF) and only significant GO terms with *P*-value <0.05 were considered. (Supplementary Tables S5, S6). Top 10 (down-regulated and up-regulated) gene-enriched GO terms were identified and shown in Figure 2. In the BP group, the down-regulated DEGs (Figure 2A) are mainly enriched in spermatogenesis, male gamete generation and spermatid development, and up-regulated DEGs (Figure 2B) are mainly enriched in regulation of cellular component movement, cell adhesion and cell migration/motility. KEGG pathway enrichment analysis of DEGs revealed that majority of the downregulated genes were associated with protein processing in endoplasmic reticulum and ubiquitin mediated proteolysis pathways (Supplementary Table S7). KEGG analysis also showed genes significantly up-regulated in KI mice involved in ECM-receptor interaction, Carbon metabolism, Focal adhesion pathways (Supplementary Table S7). Top 10 KEGG pathways (down-regulated and up-regulated) were identified by analyzing significantly DEGs which were shown as dot plots in Figures 2C,D.

### PPI Network Construction and Analysis

**FIGURE 3:**
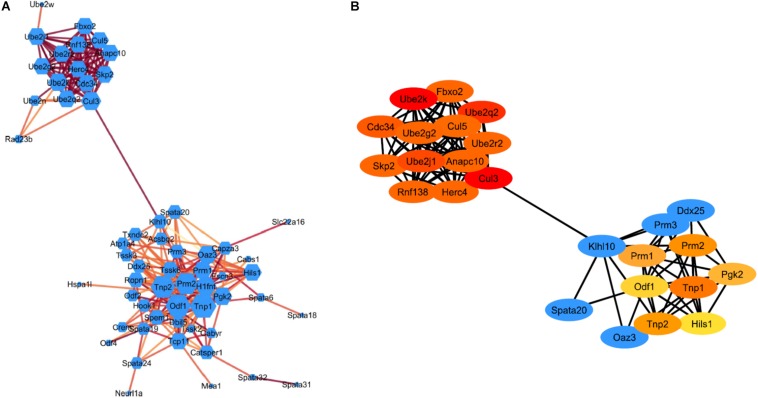
STRING protein-protein interaction network. **(A)** Visualization of the PPI network of identified down-regulated DEGs from spermatogenesis process and UPP pathway. Colored nodes represent genes/proteins. Edges represent the protein-protein associations. Node size indicates node degree value and Edge color represents stringdb score. Darker edges mean higher stringdb score **(B)** Visualization of the top 20 hub genes from the PPI network using the cytoHubba plugin in Cytoscape software. Red to yellow colored nodes represents genes with high to low PPI degree scores.

The STRING plugin app in Cytoscape was used to analyze the DEGs involved in spermatogenesis and ubiquitin-proteasome pathway (UPP) to construct a PPI network. The PPI network show 77 nodes/proteins and 247 edges/interactions, with a local clustering coefficient of 0.502 and has a PPI enrichment *P*-value <1.0e^–16^ (Figure 3A). Using network analyzer app in cytoscape we analyzed the different network parameters for each node. Based on degree value different sizes are assigned to nodes, while darker edges represent higher string database score indicating stronger degree of interactions with other proteins. Using cytoHubba plugin in Cytoscape, we identified top 20 Hub genes representing higher degree of connectivity between nodes (Figure 3B). For example, Hub genes (CUL3, UBE2K, UBE2Q2, UBE2J1) with higher degree value means highly connected network. The two modules with key hub genes (red and orange nodes) in the PPI network are interconnected to each other by KLHL10 essential for spermatogenesis process and CUL3 involved in UPP (Figure 3B). This connection is significant as it has been shown experimentally that these two proteins KLHL10 and CUL3 directly interact with each other (Wang et al., 2006).

### Validation of DEGs Using qRT-PCR

**FIGURE 4:**
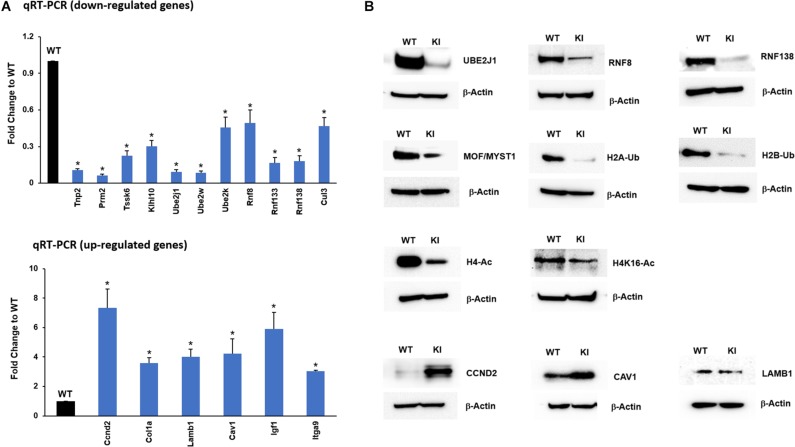
RNA-Seq data validation by qRT-PCR and Western blot. **(A)** Expression of selected up-regulated and down-regulated genes from the RNA-seq data was measured by qRT-PCR relative to actin in KI vs WT mice. Histograms represent the fold change expression between KI and WT group. Means ± SEM were determined from three sets of qRT-PCR experiments with each sample run in triplicates. *P*-values were calculated by two-tailed Students *t*-test (asterisks indicates *P* < 0.05). **(B)** Western blots showing the protein expression of UBE2J1, RNF8, RNF138, MOF/MYST1, H2A/B-Ub (Ubiquitinated Histone 2A/B), H4-Ac (Acetylated Histone 4), H4K16-Ac, CCND2, CAV1, and LAMB1 in KI and WT mice.

To further confirm and validate the transcriptome results from the RNA-seq analysis, genes relevant to spermatogenesis and UPP enriched in functional analysis were selected, and their expression levels were confirmed by qRT-PCR analysis. The results from qRT-PCR indicated that transcripts of *Tnp2*, *Prm2*, *Tssk6*, and *Klhl10* involved in spermatogenesis process and transcripts of *Ube2j1*, *Ube2k*, *Ube2w*, *Rnf8*, *Rnf138, Rnf133*, and *Cul3* that participate in UPP were downregulated in KI group consistent with RNA-seq results (Figure 4A). The upregulated genes like *Col1a*, *Lamb1*, *Cav1*, *Ccnd2*, *Itga9*, and *Igf1* crucial for focal adhesion and ECM-receptor interaction pathways were found to have increased levels of their transcripts in KI compared to WT (Figure 4A). Overall, the qRT-PCR results displayed high correlation with those obtained by RNA-Seq analysis.

### Validation of Enriched Pathway Genes Using Western Blotting

In addition to transcript levels, we wanted to confirm the protein levels of genes that were down-regulated in the qRT-PCR analysis. The protein levels of selected UPP genes UBE2J1, RNF8, RNF133, RNF138, and other prerequisite factors MOF/MYST-1, H2B-Ub, H2A-Ub, H4-Ac, and H4K16-Ac that are implicated during spermiogenesis were drastically reduced in KI compared to WT (Figure 4B) indicating that p-GRTH impacts genes which are essential in the spermatid elongation process regulating the expression of genes involved in the UPP. The protein levels of up-regulated transcripts COL1a, CCND2 were increased in KI compared to WT (Figure 4B).

### Loss of p-GRTH in KI Mice Impairs Binding of *Ube2J1* and *Rnf8* mRNA’s to GRTH Protein

**FIGURE 5:**
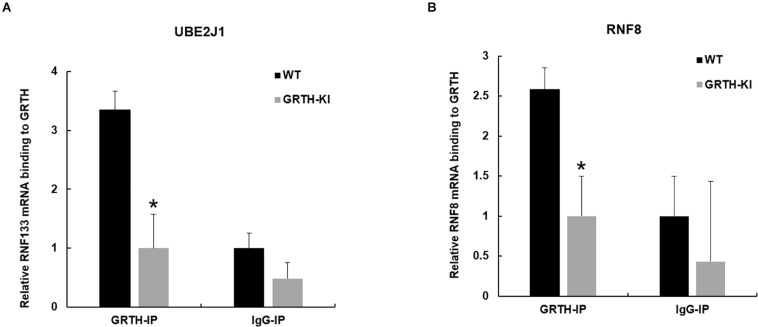
Binding of GRTH protein to *Ube2j1* and *Rnf8* mRNA in KI vs WT mice. Relative mRNA binding of *Ube2J1* **(A)**, and *Rnf8* **(B)** to GRTH protein in WT and KI mice. Statistical analysis was done using two-tailed Students *t*-test (*indicates *P* < 0.05) and data represents mean ± SEM of two independent experiments in triplicates.

Previous studies from our lab have shown that GRTH protein binds to specific germ cell mRNAs (*Tp2*, *Prm2* and *Pgk2)* at cytoplasmic sites for storage in CB and later participates in translational process (Sheng et al., 2006; Kavarthapu et al., 2013). It was of interest to learn whether GRTH associates to *Ube2J1* and *Rnf8* mRNAs, two key genes of the UPP pathway, and how these were impacted in KI mice which lacks p-GRTH. The GRTH-IP mRNA binding studies revealed that *Ube2J1* and *Rnf8* mRNAs from WT mice associated with GRTH protein and the binding is abolished in the KI mice (Figure 5).

### IHC Analysis of Genes Involved in Spermatid Elongation Process

**FIGURE 6:**
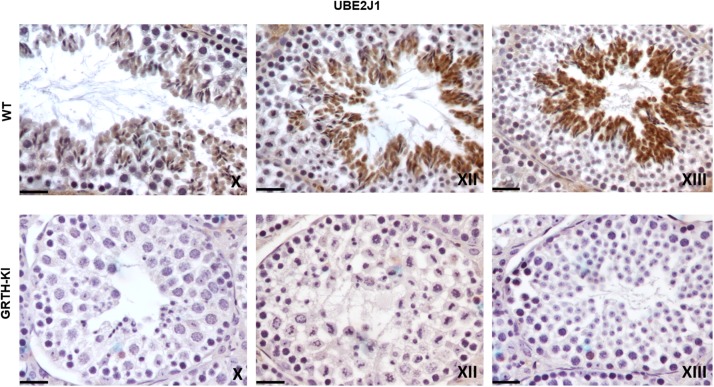
IHC analysis of UBE2J1 in seminiferous tubules of WT and KI mice. UBE2J1 expression is localized (DAB signal) specifically in ES of stage X, XII, XIII of spermatogenesis. The signal intensity was gradually increased from stage X to XIII in WT.

**FIGURE 7:**
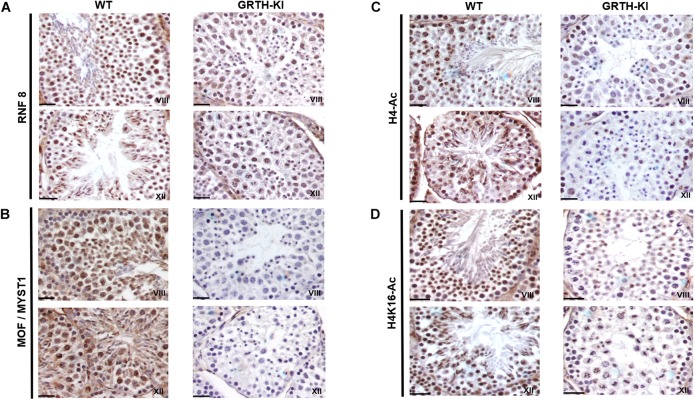
IHC analysis of RNF8, MOF/MYST1, H4-Ac and H4K16-Ac in seminiferous tubules of WT and KI mice. RNF8 **(A)**, MOF/MYST1 **(B)**, H4-Ac **(C)**, and H4K16-Ac **(D)** expression are noticed more significantly in the nucleus of RS (stage VIII) and ES (stage XII) in seminiferous tubules of WT mice while their expression are not observed in the RS of KI mice.

We selected UBE2J1, RNF8, MOF/MYST-1, H4-Ac, and H4K16-Ac genes that are relevant to spermatid elongation process for IHC analysis to elucidate their expression pattern in germ cells during spermiogenesis. In WT mice UBE2J1 expression was exclusively noticed in ES of step 10 and gradually intensified in condensing spermatids of steps 12 to 13 of spermiogenesis (Figure 6). There was no expression of UBE2J1 in KI mice compared to WT mice (Figure 6). RNF8 and MOF expression was observed in spermatocytes, RS and ES of WT mice (Figure 7A). While their expression was abundant in round spermatids of WT mice, in KI mice was drastically reduced. As the RS elongate and chromatin starts condensing in later stages (stage XII), RNF8 expression was observed in the nucleus of ES of step 12 in WT mice. While in KI mice seminiferous tubule at stage XII, we found RS that fail to elongate and the RNF8 signal in these germ cells was very weak (Figure 7A). MOF/MYST1 also showed similar expression patterns with much higher signals in nucleus of spermatocytes, RS and ES of WT mice while merely low to negligible expression in germ cells of KI mice (Figure 7B). Acetylation of H4 observed in the nucleus of round spermatids was dramatically reduced in KI mice compared to WT (Figure 7C). Similar expression pattern was observed for the specific acetylation of H4 at K16 residue (Figure 7D).

## Discussion

In this study, we examined genome-wide transcriptome profiles of germ cells isolated from GRTH-KI and WT mice testis using RNA-Seq. We used homozygous KI mice with R^242^H mutation in GRTH gene with resulting loss of p-GRTH to further understand its role in spermatid development during spermiogenesis by analyzing the DEGs from RNA-Seq. Previously we have shown that homozygous KI mice are infertile and lack ES and mature sperm due to arrest of round spermatids of step 8 that fail to elongate in the process of spermatogenesis. Absence of p-GRTH in KI mice had a profound impact on spermatid development indicated by abnormal acrosome formation in round spermatids which later undergo apoptosis forming multinucleated giant spermatids. The RNA-Seq analysis of transcriptome of germ cells in KI vs WT mice revealed DEGs related to ECM-receptor interaction and focal adhesion pathways were up-regulated. Genes like collagen, integrins, laminins and others involved in focal adhesion and ECM-receptor interaction pathways play a crucial role in junction dynamics in the seminiferous epithelium regulating cell-matrix interactions and cell-cell adhesion and cell migration (Siu and Cheng, 2004). We believe that these facilitate aggregation/clumping of round spermatid together and formation of giant multi-nucleated germ cells as observed in our histological studies in KI mice (Kavarthapu et al., 2019). We found several DEGs related to spermatogenesis, spermatid development and UPP were downregulated. Of note is that in germ cells of KI mice there was a dramatic reduction in UPP proteins (UBE2J1 and RNF8) essential for chromatin remodeling during spermiogenesis. This study using the KI mice model demonstrated that loss of p-GRTH impairs UBE2J1 and RNF8 dependent histone modifications hampering initial stages of spermatid elongation process.

During spermiogenesis, round spermatids go through 16 steps of development with subsequent characteristics variations in cell morphology and nuclear condensation as characterized by development of elongating, condensing, and condensed spermatids. RS of steps 1–8 possess round-shaped cells with preponderant nuclei packed with basic histone proteins and are in a state of active transcription. The step 8 RS exhibit distinct cap shaped acrosome (Russell et al., 1990; Hecht, 1998). Histone acetylation that leads to histone replacement later occurs in RS of step 8 prior to the initiation of nuclear elongation (Hazzouri et al., 2000; Shirakata et al., 2014). ES of steps 9–11 show hyperacetylation of histones, nuclear elongation and concurrent extension of the acrosome. Replacement of histones by TNP1, TNP2 and subsequently by PRM1 and PRM2 occurs in the condensing spermatids of steps 12–14 (Bao and Bedford, 2016). Condensed spermatids of steps 15,16 also known as spermatozoa display a typical hook type morphology and are ready to be released into the lumen of seminiferous tubules. TSSK6, HSP90 and γH2AX are among other genes shown to play an important role in chromatin remodeling and condensation of spermatids during spermiogenesis (Jha et al., 2017). In this study, functional analysis of DEGs showed that genes (*Tnp1/2, Prm1/2, Tssk3/4/6, Hsp90, Klhl10*) mainly associated with biological process, such as spermatogenesis, male gamete generation and spermatid development were significantly down-regulated in KI mice. This is consistent with previous studies demonstrating complete loss of TNP2 and PRM2 and TSSK6 protein expression in KI compared to WT (Kavarthapu et al., 2019). Further, the KEGG pathways analysis of down-regulated DEGs are mainly enriched in protein processing in endoplasmic reticulum and ubiquitin-proteasome pathways. Further, using STRING database we analyzed and constructed PPI network of down-regulated DEGs showing two significant modules with key hub genes belonging to spermatogenesis process and UPP. Moreover, these two modules are interconnected by KLHL10 and CUL3 proteins. CUL3 (Cullin3) is a Core component of cullin-RING-based E3 ubiquitin-protein ligase complex which interacts with substrate-specific adapter KLHL10 and functions specifically in the testis to mediate protein during spermiogenesis (Wang et al., 2006). Mutations in KLHL10 gene have been associated with oligospermia in some infertile males (Yatsenko et al., 2006). Haploinsufficiency of KLHL10 causes infertility in male mice devoid of ES due to failure of RS elongation after step 8 precisely similar morphology observed in our KI model (Yan et al., 2004; Wang et al., 2006).

**FIGURE 8:**
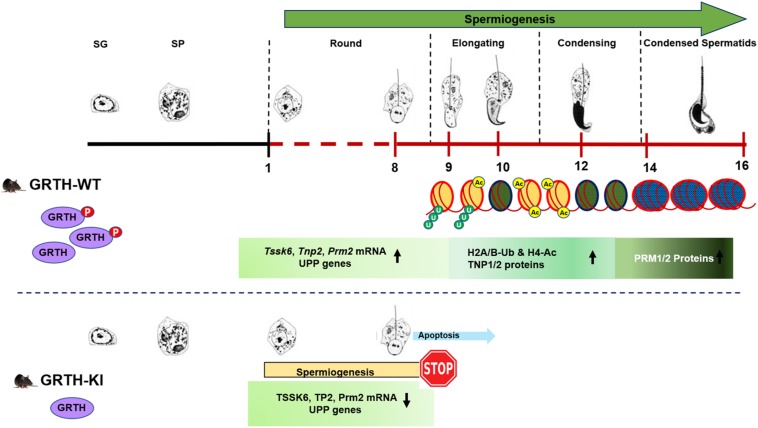
Role of p-GRTH in histone ubiquitination and acetylation essential for round spermatid during spermiogenesis. Schematic diagram showing progression of mice spermiogenesis where germ cells undergo 16 different steps of development. In WT mice during the process of spermiogenesis in RS we observed increased and stable expression of Tssk6, Tnp2, Prm2, and UPP mRNAs until ready for translation in step 9 of RS. During spermatid elongation histones undergo ubiquitination (H2A/B-Ub) and acetylation (H4-Ac) resulting in their removal and replacement with transition proteins and protamines making the chromatin more compact/condensed. Of note is that in KI mice loss of p-GRTH has direct impact on expression of UPP genes required for ubiquitination and subsequent acetylation which impairs round spermatids to elongation.

Histone ubiquitination and acetylation play a crucial role in chromatin remodeling essential for development of spermatids during spermiogenesis (Baarends et al., 1999a, b; Rathke et al., 2014; Sheng et al., 2014). H2A is highly ubiquitinated in the XY bodies of pachytene spermatocytes in meiotic phase I. Ubiquitinated H2A and H2B are also abundantly found in ES and play important roles in chromatin remodeling during spermiogenesis (Baarends et al., 1999a; Sheng et al., 2014). Histone hyperacetylation mediated by specific histone acetyltransferases (HATs) like MOF/MYST1 and TIP60 during spermiogenesis facilitate histone replacement (Taipale et al., 2005; Thomas et al., 2007). E2 ubiquitin-conjugating enzyme UBE2J1 and ubiquitin E3 ligase RNF8 are part of an E2/E3 ubiquitin ligase complex shown to play an important role in spermatid development during spermiogenesis (Lu et al., 2010; Koenig et al., 2014). UBE2J1 KO mice are sterile with major defect in spermatid differentiation, resulting in male sterility acting as a key player in the elongation of spermatids (Koenig et al., 2014). Several testis-specific E3 ubiquitin ligases are known to be involved in wide-ranging molecular events during spermiogenesis including organelle turnover, protein turnover and quality and chromatin remodeling (Hou et al., 2012). RNF8 mediated H2A/H2B ubiquitination shown to be essential for histone hyperacetylation and important for histone removal at post-meiotic stage of germ cells (Lu et al., 2010). Furthermore, it has been shown that RNF8-dependent histone ubiquitination controls H4K16 acetylation by regulating association of MOF on the chromatin, which could be a crucial step for histone removal in the early ES (Lu et al., 2010; Ma et al., 2011). In our study, genes related to the UPP were significantly down-regulated (Figure 4A). Although there was no differential expression of RNF8 in our RNA-Seq data, we found marked reduction in transcript levels of RNF8 and UBE2J1, and their proteins are barely detectable in KI mice. This consequently led to impair ubiquitination of H2A and H2B which is evident by the negligible expression of H2A-Ub and H2B-Ub proteins in Western blots. Further, the marked reduction in the association of p-GRTH protein with *Ube2j1* and *Rnf8* mRNA’s may affect their translation as p-GRTH is known to associate with polyribosomes of germ cell specific genes during spermiogenesis (Sheng et al., 2006). We also found reduced expression of MOF in KI mice which facilitates acetylation of H4 during initial stages of spermatid elongation. Further, as expected these low levels of MOF hampered the acetylation of H4 and specifically of H4K16 which is evident by the significant reduction of their expression in the nucleus of RS and ES in our IHC analysis. Taken together our studies indicate that the complete impairment of spermatid development in KI mice lacking p-GRTH, is linked to the deficit on the expression of UBE2J1 and RNF8. These, in turn consequently disrupted the chain of events involving H2A/H2B ubiquitination and H4 acetylation for subsequent histone removal and replacement with transition proteins followed by protamines during spermiogenesis (Figure 8).

## Data Availability Statement

The RNA-Seq raw data files (KI and WT samples) in this study have been submitted to the NCBI (https://www.ncbi.nlm.nih.gov/geo) Gene Expression Omnibus (GEO accession number GSE145047). All other RNA-Seq results analyzed during this study are included in this article and its supplementary files.

## Ethics Statement

The animal study was reviewed and approved by National Institute of Child Health and Human Development Animal Care and Use Committee.

## Author Contributions

RK and MD conceived and planned the experiments. RK and RA performed the experiments, analyzed the data. MD and RK discussed the results and wrote the manuscript. AS and JS contribute with RNA-Seq data analysis.

## Conflict of Interest

The authors declare that the research was conducted in the absence of any commercial or financial relationships that could be construed as a potential conflict of interest.

## Footnotes

1 https://www.ncbi.nlm.nih.gov/geo

2 http://www.partek.com/partek-flow/

3 https://string-db.org/

4 http://david.abcc.ncifcrf.gov/
