# Supplementary material for: Linking Phospho-Gonadotropin Regulated Testicular RNA Helicase (GRTH/DDX25) to Histone Ubiquitination and Acetylation Essential for Spermatid Development During Spermiogenesis
Source: Front Cell Dev Biol. 2020 May 15;8:310. doi: 10.3389/fcell.2020.00310 (PMC7242631; doi:10.3389/fcell.2020.00310)
Supplement: Supplementary file 1 [file Table_1.DOCX]

**List of Primers used for Validation of DEGs using qRT-PCR**

| **Primer Name** | **Primer Sequence 5’--- 3’** |
| --- | --- |
| **KI-F1** | AGA ACG GCA TCA AGG TGA AC |
| **KI-R1** | GCC TCC CCA CAG TAA CGA C |
| **KI-F2** | GAT ACC TAA GTT GGC TAG GTA TCC TGA G |
| **KI-R2** | ATA GTT GCC CAA GCT GCT ACC CCA GTT CCA |
| **Rnf8 F** | GGA TGA AAC GGA AAG TAG AGT GTC |
| **Rnf8 R** | CAT GAC AGT CTC TTT GCT CTC CGT TC |
| **Rnf138 F** | GGA TAC AAG TTC TTC TGG GCA |
| **Rnf138 R** | GAC TCT TCC ACA GCA GTT TGA T |
| **Rnf133 F** | GGT AAC ATC AAA GCA TAT TTC ACC |
| **Rnf133 R** | GGA TTA ACT TCT TCA TCC CCT TC |
| **Ube2w F** | GTC AAT GCA GAA ACG ACT ACA |
| **Ube2w R** | GAG GAG AGT CAA AAG GGT ATC G |
| **Ube2j1 F** | CAT GGA GCA TAA GGA CAG CCT |
| **Ube2j1 R** | GCA ACA GGA CAT CCT TCA TGG |
| **Ube2k F** | GAA GAG CGA GGA GGT CCG GT |
| **Ube2k R** | GCA GCC GCC AAC AGC GCT TG |
| **Itga9 F** | ACA CGC GCT GGG TCC TCG TG |
| **Itga9 R** | GCC ATG TCC AGC TCG GTG CAT |
| **Ccnd2 F** | GGA TGC TAG AGG TCT GTG AGG A |
| **Ccnd2 R** | GCT TCA CAG AGT TGT CGG TG |
| **Col1a1 F** | GCT GGT CCT AAG GGA GAG C |
| **Col1a1 R** | GGT CCA GCA GCA CCA ACA GC |
| **Lamb1 F** | GAA GGA ATG GTT CAC GGG CAC |
| **Lamb1 R** | GAC ACG AGC TGG AAT GTT CAT |
| **Igf1 F** | GCT TTT ACT TCA ACA AGC CCA C |
| **Igf1 R** | GTA CAT CTC CAG TCT CCT CAG |
| **Cav1 F** | GAC GTG GTC AAG ATT GAC TTT G |
| **Cav1 R** | GAT GAG TGC CAT TGG GAT GC |
| **Cul3 F** | GATTCAAACAGTTGCAGCCAAACAAG |
| **Cul3 R** | CTGGACTTGGTAAGAATCGAGCCT |
| **Tnp2 F** | GCTCTAGCTCCAGCCCCAGC |
| **Tnp2 R** | CTTGTATCTTCGCCCTGAG |
| **Prm2 F** | GGCAAGGGCTGAGCCCAGAGC |
| **Prm2 R** | TCGGGATCTTCTGCAGCCTCTGC |
| **Tssk6 F** | CCGCGAAGTCGCCGCGCGCCACTG |
| **Tssk6 R** | TCTCTCTTCTCTTTGCGCCCCTCC |
| **Klhl10 F** | GTC TCA ACA CTG CTG AAC G |
| **Klhl10 R** | CAT ATG TAG ACC TTC CCA TAG AG |
| **β-actin F** | AGGCATTGCTGACAGGATGCAG |
| **β-actin R** | AGCACTTGCGGTGCACGATG |
