# Supplementary material for: Linking Phospho-Gonadotropin Regulated Testicular RNA Helicase (GRTH/DDX25) to Histone Ubiquitination and Acetylation Essential for Spermatid Development During Spermiogenesis
Source: Front Cell Dev Biol. 2020 May 15;8:310. doi: 10.3389/fcell.2020.00310 (PMC7242631; doi:10.3389/fcell.2020.00310)
Supplement: Supplementary file 2 [file Table_2.DOCX]

**List of primary antibodies used for Western blots**

| **Antibody Name** | **Company** | **RRID No.** |
| --- | --- | --- |
| UBE2J1 | Santa Cruz Biotechnology | AB_10611604 |
| RNF8 | Santa Cruz Biotechnology | AB_11155731 |
| RNF138 | Santa Cruz Biotechnology | AB_10610897 |
| MOF/MYST1 | Santa Cruz Biotechnology | AB_1126484 |
| H2A-Ub | Millipore | AB_11214408 |
| H2B-Ub | Millipore | AB_1587119 |
| H4-Ac | Cell Signaling Technology | AB_1118600 |
| H4K16-Ac | Cell Signaling Technology | AB_2798923 |
| CCND2 | Cell Signaling Technology | AB_2070685 |
| CAV1 | Santa Cruz Biotechnology | AB_2275453 |
| LAMB1 | Santa Cruz Biotechnology | AB_627866 |
| β-ACTIN | Santa Cruz Biotechnology | AB_1119529 |
